# Epidemiological surveys of, and research on, soil-transmitted helminths in Southeast Asia: a systematic review

**DOI:** 10.1186/s13071-016-1310-2

**Published:** 2016-01-27

**Authors:** Julia C. Dunn, Hugo C. Turner, Aung Tun, Roy M. Anderson

**Affiliations:** London Centre for Neglected Tropical Disease Research, London, UK; Department of Infectious Disease Epidemiology, School of Public Health, Faculty of Medicine, Imperial College London, London, UK; Worm Free Myanmar Kids Program, Tun Khit Foundation, Yangon, Myanmar

**Keywords:** Soil-transmitted helminths, Southeast Asia, Systematic review, Neglected tropical diseases, Mass drug administration, Monitoring and evaluation

## Abstract

Soil-transmitted helminth (STH) infections of humans fall within the World Health Organization’s (WHO) grouping termed the neglected tropical diseases (NTDs). It is estimated that they affect approximately 1.4 billion people worldwide. A significant proportion of these infections are in the population of Southeast Asia. This review analyses published data on STH prevalence and intensity in Southeast Asia over the time period of 1900 to the present to describe age related patterns in these epidemiological measures. This is with a focus on the four major parasite species affecting humans; namely *Ascaris lumbricoides*, *Trichuris trichiura* and the hookworms; *Necator americanus* and *Ancylostoma duodenale*. Data were also collected on the diagnostic methods used in the published surveys and how the studies were designed to facilitate comparative analyses of recorded patterns and changes therein over time. PubMed, Google Scholar, EMBASE, ISI Web of Science, Cochrane Database of Systematic Reviews and the Global Atlas of Helminth Infections search engines were used to identify studies on STH in Southeast Asia with the search based on the major key words, and variants on, “soil-transmitted helminth” “*Ascaris*” “*Trichuris*” “hookworm” and the country name. A total of 280 studies satisfied the inclusion criteria from 11 Southeast Asian countries; Brunei, Cambodia, Indonesia, Lao People’s Democratic Republic (Lao PDR), Malaysia, Myanmar, Philippines, Singapore, Thailand, Timor-Leste and Vietnam. It was concluded that the epidemiological patterns of STH infection by age and species mix in Southeast Asia are similar to those reported in other parts of the world. In the published studies there were a large number of different diagnostic methods used with differing sensitivities and specificities, which makes comparison of the results both within and between countries difficult. There is a clear requirement to standardise the methods of both STH diagnosis in faecal material and how the intensity of infection is recorded and reported in future STH research and in monitoring and evaluation (M&E) of the impact of continuing and expanding mass drug administration (MDA) programmes.

**Electronic supplementary material:**

The online version of this article (doi:10.1186/s13071-016-1310-2) contains supplementary material, which is available to authorized users.

## Background

Soil-transmitted helminth (STH) infections belong to the so called neglected tropical diseases (NTDs) that affect human populations in poorer regions of the world [1]. Their presence is a typical marker of poverty where access to sanitation and clean water is limited and, concomitantly, standards of hygiene are low [2–5]. There are four main species of STH; namely, *Ascaris lumbricoides* (roundworm), *Trichuris trichiura* (whipworm) and the hookworms (*Ancylostoma duodenale* and *Necator americanus*) [6].

It is estimated that over 1.4 billion people are infected with STHs [2, 7, 8]. According to a study by Pullan et al. [8], the highest number of STH infections occurs in Asia, where the People’s Republic of China and India have the greatest concentration of people infected with intestinal worms. Southeast Asia is the region with the highest reported prevalences of STH infection in recent decades [8, 9].

The countries of Southeast Asia have various attributes that contribute to the continually high prevalence of STH. For example, most Southeast Asian countries have a tropical and moist climate, which is ideal for the survival of STH eggs/larvae in the environment [10]. This environment acts to promote infection within the human population [10, 11]. Socioeconomic factors such as lack of adequate water resources, sanitation and poor hygiene practices have repeatedly been proven to be related to high STH prevalence within a community [5] since transmission of *A. lumbricoides* and *T. trichiura* occurs via the faecal-oral route [6]. Several countries within Southeast Asia are amongst the poorest in the world, without adequate water and sanitation infrastructure [10, 12] and, therefore, the parasites prosper in such environments [13, 14].

The goal set by the World Health Organization (WHO) for STH control by 2020 is to reduce morbidity from STH in preschool-aged (pre-SAC: 2-5 years) and school-aged children (SAC: 5-14 years) to a level below which it would not be considered a public health problem [15]. Similarly, the target set by the 2012 London Declaration on NTDs, is to achieve preventive chemotherapy (PCT) coverage of 75 % of all pre-SAC and SAC at risk of STH by 2020 [16, 17]. To meet this goal, Southeast Asian countries that are endemic for STH have been conducting mass drug administration (MDA) campaigns [13, 18], treating pre-SAC and SAC in affected areas with antihelminthic drugs such as albendazole and mebendazole at regular intervals [19, 20]. The current goals and objectives set by the WHO focus on reducing morbidity in pre-SAC and SAC, the age groups most commonly and most severely affected by two of the major STH infections; namely, *A. lumbricoides* and *T. trichiura* [15]. Hookworm is found at the highest intensities in adults, and hence, its abundance is not greatly affected by only treating pre-SAC and SAC [21–23]. At present, there is a growing interest in investigating the feasibility of interrupting the transmission of STH by broadening the range of ages targeted for treatment and increasing coverage in all age groups [24–27].

To evaluate the impact of MDA, comprehensive epidemiological studies need to be conducted periodically to measure changes in the prevalence and intensity of each STH species over time [28]. With the increasing focus on the effect that SAC-targeted MDA has on prevalence and intensity of STH across all ages [22, 29], ideally monitoring and evaluation (M&E) of control impact should be based on epidemiological studies that are community wide. Also, to be able to compare progress between different regions and countries, the methods and design of M&E should be standardised in terms of the diagnostic method used and the prevalence plus intensity measures made in the target population [30].

In this paper we review published epidemiological studies of STH in the Southeast Asia countries of Brunei, Cambodia, Indonesia, Lao People’s Democratic Republic (Lao PDR), Malaysia, Myanmar, Philippines, Singapore, Thailand, Timor-Leste and Vietnam. The overall aim is to evaluate past STH publications from studies conducted in Southeast Asia and to help point to the ideal study design for the M&E of control programme impact.

## Review

This systematic review was developed in line with the Preferred Reporting Items for Systematic Reviews and Meta-Analyses (PRISMA) guidelines (see Checklist in Additional file 1).

### Selection criteria

We include all published studies in English in which the prevalence and/or intensity of STH infection was measured in the Southeast Asia countries of Brunei, Cambodia, Indonesia, Lao PDR, Malaysia, Myanmar, Philippines, Singapore, Thailand, and Vietnam (the members of the Association of Southeast Asian Nations - ASEAN), within the period of January 1^st^, 1900 to July 2015. Timor-Leste was also included due to its proximity to the other Southeast Asian countries and since it is often included in public health analyses concerning Southeast Asia [8,13]. No studies were found concerning STH in Brunei. Observational and intervention studies were eligible for inclusion. We excluded studies that had the following criteria: (i) studies that did not report prevalence values for each STH separately (that just recorded prevalence of any STH); (ii) studies where the participants were selected from hospital in-patients; (iii) studies where the participants were not permanent residents of the specific country (e.g. refugees or migrants); (iv) duplicate publications or extension of analysis from an original study; and (v) studies where the full publication could not be obtained.

### Search strategy and methodology

We identified published studies using automated database searches of EMBASE (1947 to July 2015), ISI Web of Science (1900 to July 2015), the National Library of Medicine’s PubMed (1900 to July 2015) and further manual searching was done using Google Scholar, the Cochrane Database of Systematic Reviews (CDSR) and the website of the Global Atlas of Helminth Infections (GAHI - http://www.thiswormyworld.org/). We employed the following terms and variations on these terms: *STH*, or *soil-transmitted helminth*, or *Ascaris*, or *Trichuris*, or *hookworm*, or *Ancylostoma*, or *Necator*, or *deworm*. A full list of the search terms is provided in Additional file 1. We also searched the Global Neglected Tropical Diseases database [31] for data collected on STH from studies conducted in Southeast Asia but did not find any results.

**Fig. 1:**
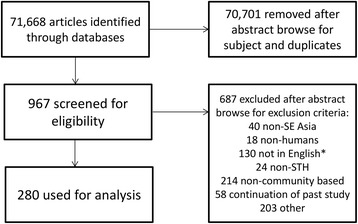
Decision tree outlining the inclusion and exclusion criteria of the identified studies. *These papers could not be properly screened due to being published in non-English language journals and likely include studies in non-included countries such as Japan and South Korea

Identified studies were exported into EndNote X6 (Thomson Reuters, New York, USA) for management. The abstracts of the studies were reviewed against the inclusion and exclusion criteria. The literature selection process is outlined in Fig. 1. Ultimately, 280 studies were identified that met the inclusion criteria, a full list of the included studies is provided in Additional file 2.

### Data extraction

Data extraction included country name, study area, year the study was published, year the study was conducted, type of study (cross-sectional or longitudinal/cohort), sample size, age of participants, prevalence of each STH, intensity of each STH, and diagnostic methods employed. If the publication did not state which year the study took place, the year of publication was used instead. Also, if the study was conducted over a range of years then the latest year of the stated range was used. For plotting the age distributions, the mid-point of the stated age range was used.

## Results and discussion

### All identified studies

**Fig. 2:**
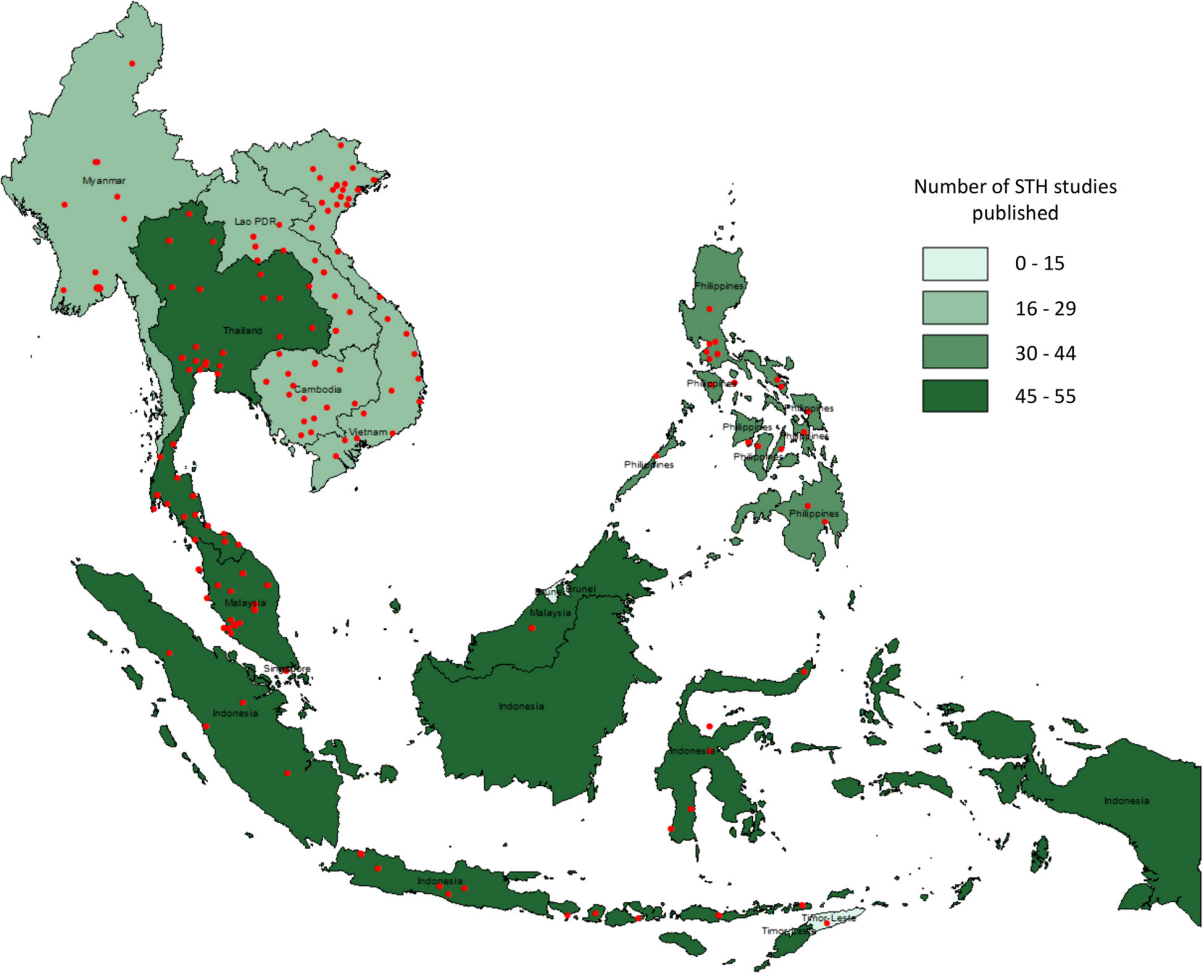
Map of identified STH studies in Southeast Asia. Red circles indicate the location of published STH studies

A total of 280 studies were identified that met the inclusion criteria (Fig. 1). The breakdown of the number of studies by country is provided in Table 1. Figure 2 illustrates the geographical distribution of study areas covered in the selected publications which met the inclusion criteria.

**Table 1:** Breakdown of the identified studies

Country	Number of studies	Number of prevalence studies that measured intensity	Number of studies with prevalence by age groups	Number of studies with age groups and intensity	Number of studies with full age distribution and intensity
Cambodia	22	1	4	0	0
Indonesia	48	9	30	6	4
Lao PDR	26	2	10	1	1
Malaysia	51	11	20	6	1
Myanmar	22	4	9	4	4
Philippines	37	7	13	1	1
Singapore	2	0	1	0	0
Thailand	55	4	13	4	4
Timor-Leste	1	0	0	0	0
Vietnam	20	5	5	2	2
TOTAL	280^a^	43	105	24	17

^a^Includes two studies that had components in two different countries, and one study that had components in three different countries

**Fig. 3:**
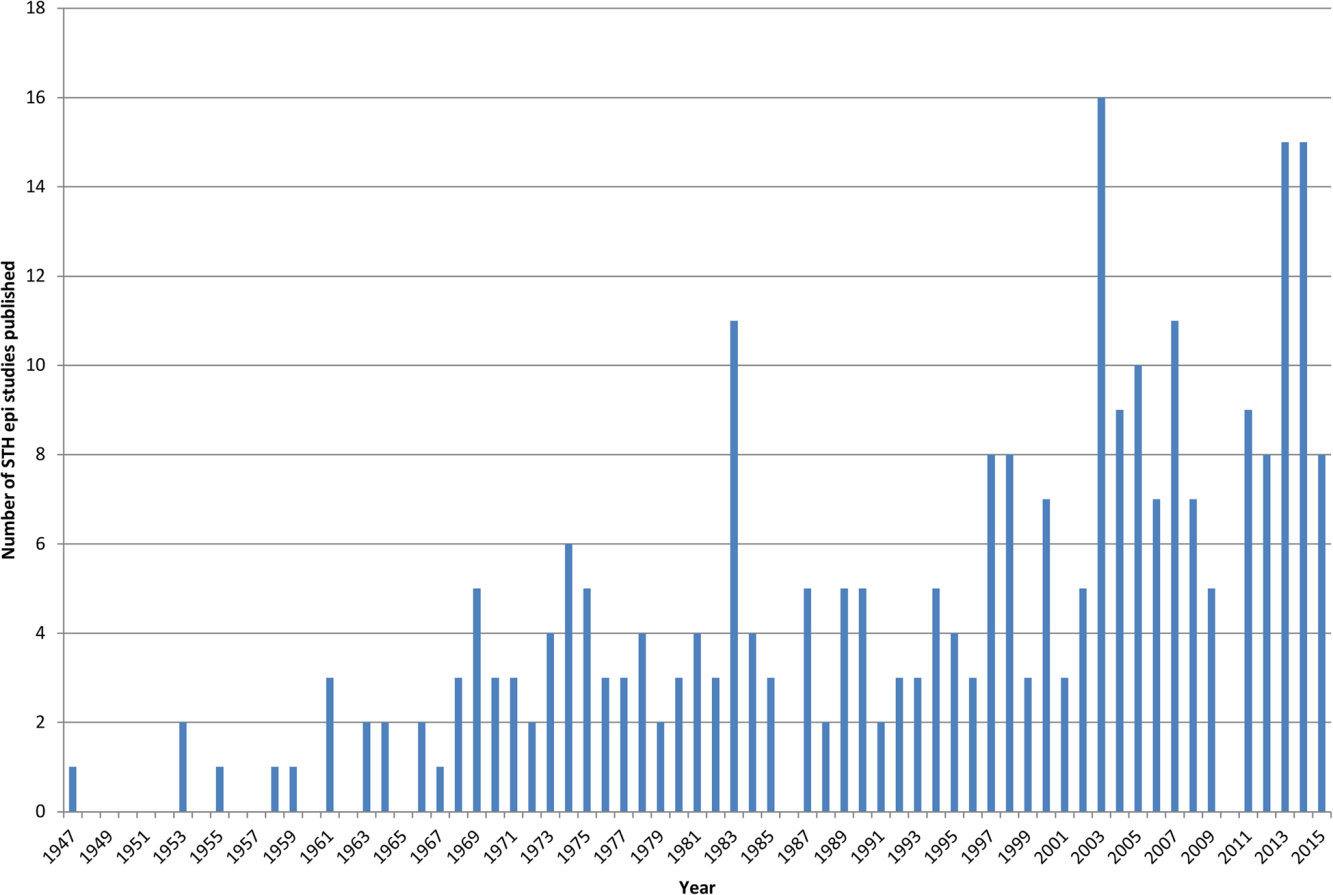
Number of identified studies published by year

The largest proportion of the identified studies were conducted in Thailand (55 studies, 20 %), followed by Malaysia (51 studies, 18 %) and Indonesia (48 studies, 17 %). Timor-Leste and Singapore had the fewest studies (one and two studies respectively). Two studies included data from two countries [32, 33] and one study included data from three countries [34]. Most of the studies identified were of a cross-sectional design (266 studies, 95 %) and the remaining studies were longitudinal (14 studies, 5 %). Figure 3 illustrates the distribution of the identified studies across time – the first study being undertaken in 1947. The number of published studies has increased steadily since 1947 to the present. In 2003 there was a marked increase in STH studies published perhaps due to an increased focus on the NTDs globally as a result of WHO guidance [18]. Another recent spike in the number of STH studies published coincides with the London Declaration on NTDs in 2012 [17]. Both of these events signalled an increase in funding and materials to combat STH, including for example the donation of albendazole tablets by GSK. The surge in published studies around these times, plus the longer term increasing trend, suggests a growing interest in STH control.

A large proportion of the selected studies measured STH prevalence and intensity from more than one study area within the same publication. Hookworm was, marginally, the most studied STH (256 studies), followed by *A. lumbricoides* (251 studies) and *T. trichiura* was the least studied (241 studies). This went against the expectation that hookworm would be the least studied of the STHs in Southeast Asia, as the climate of sub-Saharan Africa is more suitable for hookworm transmission, whereas the warm and humid climate of Southeast Asia is ideal for *A. lumbricoides* and *T. trichiura* [9,10]. Conversely, it seems that most identified studies were inclusive of all STH species.

### Diagnostic methods

**Fig. 4:**
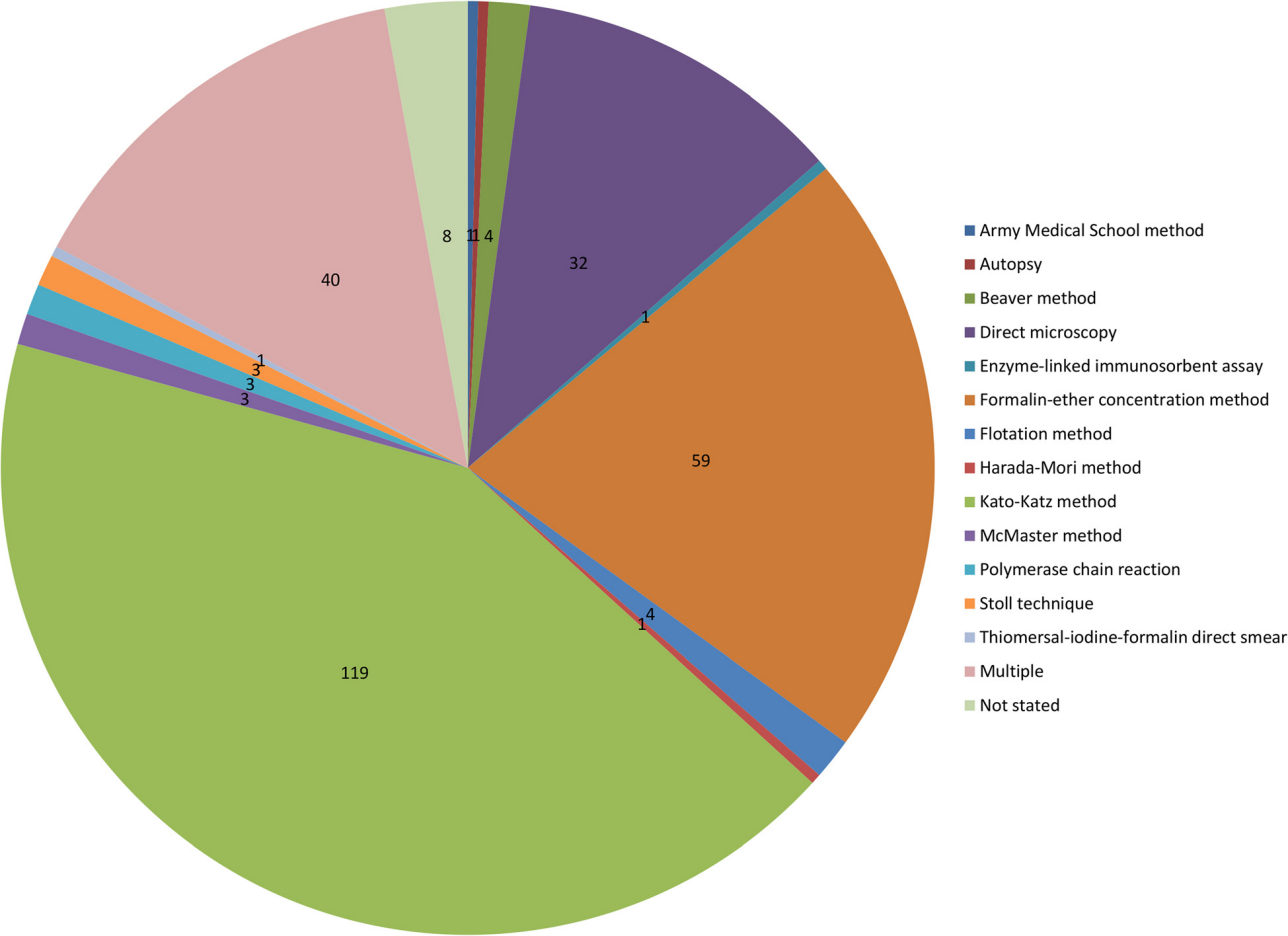
Number of studies that used each STH diagnostic method. Multiple = studies that reported using multiple methods of diagnosis

The differences in the method of STH diagnosis and quantification of intensity used across the selected studies made it difficult to compare studies. For example, STH prevalence was measured by 13 different methods (Fig. 4). Of the 280 studies, 40 reported using more than one method for diagnosing STH infection, whilst eight studies did not report the method used at all. The specificity and sensitivity of the different methods of STH diagnosis have been analysed in a number of publications and have been found to vary widely [30, 35–37]. Therefore, it can be inferred that the accuracy of the prevalence and intensity results in STH studies also varies over time. However, it is difficult to quantify this due to the lack of standardised procedures.

The Kato-Katz technique was the most frequently used method of STH diagnosis (128 studies (45.7 %), including studies that used multiple methods). The proportion of studies using each type of diagnostic method has changed over time, with the proportion of studies using Kato-Katz and the formalin-ether concentration (FEC) method increasing since the 1980s. The current decade has also seen the first studies using more advanced molecular and immunological diagnostic tools such as the polymerase chain reaction (PCR) [38–40] and enzyme-linked immunosorbent assay (ELISA) [41]. In each decade there was a small proportion of studies that do not specify which method the authors used to diagnose STH infection.

There was also variation in how the diagnostic methods were applied. For example, variation between studies is apparent where the Kato-Katz method was used as the primary diagnostic method. Studies varied in how many Kato-Katz thick smears were prepared from each participant, how many times these slides were read (for quality control) [42] and whether or not readings were by the same operator or different people. Out of the 128 studies that used Kato-Katz, 97 (75.8 %) did not clearly state that any repetition had been made, 11 (8.6 %) studies stated that they prepared/read Kato-Katz slides once, 19 (14.8 %) studies in duplicate and one study in the Philippines [43] read Kato-Katz slides six times.

### Prevalence and intensity metrics

Most control programmes use prevalence as their main epidemiological indicator, as advised by the WHO [15, 19]. However, prevalence as an indicator [21, 44, 45] is far from ideal given the highly non-linear relationship between this measure and the average intensity of infection when parasite distributions of worms or eggs per gram (EPG) output per host is aggregated (negative binomial) in form [46].

**Fig. 5:**
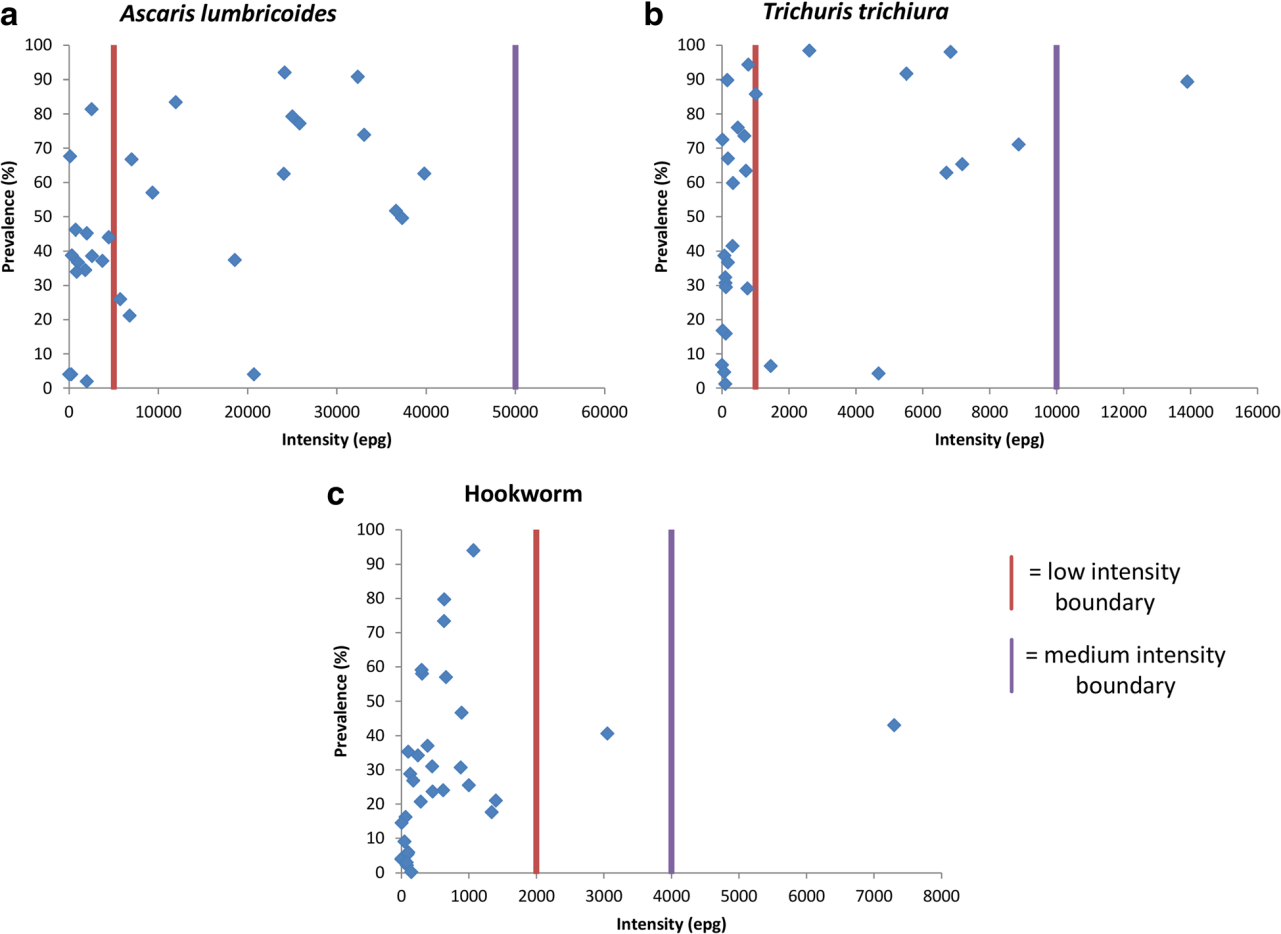
STH prevalence (% infected) plotted against the average intensity for studies that measured intensity in eggs per gram (EPG) of faeces. Red line indicates the upper threshold for low intensity, purple line indicates the upper threshold for medium intensity, above purple line is high intensity as defined by the WHO [15]. **a** = Ascaris lumbricoides, (**b**) = Trichuris trichiura, (**c**) = Hookworm

Figure 5 shows overall STH prevalence plotted against average intensity for the studies that measured intensity by EPG counts. There are clearly discernible relationships between STH prevalence and average intensity displayed in these plots. Prevalence is non-linearly related to average intensity where the former changes rapidly at low intensities, but slowly at high intensities. The importance of this relationship lies in the observation that large changes in intensity, possibly caused by the effects of MDA, are not well measured by changes in prevalence. Therefore, M&E for MDA programmes must be based on intensity. The precise relationship between the two epidemiological measures is determined by the magnitude of the negative binomial aggregation parameter *k* (which varies inversely with the degree of aggregation). For high aggregation prevalence plateaus well below 100 %, while for low degrees of aggregation of worms it quickly saturates to high prevalence figures [46]. The heterogeneity displayed in this non-linear relationship may be due in part to the inclusion of data from different settings, countries and decades on the same graph. The vertical lines on Fig. 5 indicate the boundaries of the intensity groupings defined by the WHO as low, medium and high [15]. Note that for values within one intensity classification (low, medium or high) the prevalence of infection varies widely. This reflects differing degrees of worm aggregation within the various human communities studied. For example, the prevalence values for hookworm in the low intensity group (low intensity group mean EPG 414.42) ranged from close to 0 to 94 %.

As specified in the inclusion criteria, all of the studies selected reported at least one prevalence value for a species of STH. Of these 280 studies, only 43 (15 %) also measured STH intensity of infection in the study participants. Table 1 details the number of studies that measured intensity of STH in participants. Similar to prevalence, STH intensity was measured using a variety of different methods between studies. In 29 studies intensity was measured using the indirect method of mean EPG of faeces. Eight additional studies used EPG but presented the geometrical means only. Five studies used the more direct method of mean worm burden based on worm expulsion in faeces post chemotherapy. A single study used eggs per millilitre (EPM) of faeces as the measure of intensity.

### Age distributions of prevalence and intensity of infection

The current focus of STH control, determined by the goals set by the WHO [15], is reducing morbidity in pre-SAC and SAC, the age groups that suffer the highest morbidity from heavy *A. lumbricoides* and *T. trichiura* infection [18, 47]. Consequently, most studies are focussed on STH infection in these age groups. However, recent mathematical model-based studies of STH transmission dynamics [23, 29, 44, 48, 49] have helped focus attention onto the burden of infection in adults and how it affects overall STH transmission in a given community. These studies concluded that in many settings, transmission could not be interrupted by only SAC-focussed MDA, this is especially true for hookworm where prevalence and intensity is highest in the adult age groups [21,50]. Therefore, in many instances morbidity control will not lead to elimination, as adults will not be treated and transmission will not be broken. To break transmission, country MDA programmes may have to adapt to include all age groups. As such, there is a need for comprehensive epidemiological studies measuring prevalence and intensity across all age groups.

Table 2 presents the details of the 17 studies that measured STH prevalence and intensity of infection in all age groups within a community (6 % of the total number of identified studies). The small number of these studies is indicative of the lack of comprehensive epidemiological M&E of control impact of MDA on STH in the Southeast Asia region. Cambodia, Singapore and Timor-Leste did not have any studies that fit these criteria. Even within these 17 studies, there is substantial variation in how prevalence and intensity are measured and summarised (Table 2).

**Table 2:** Details of the studies that measured intensity across all ages

First Author	Year	Country	Sample size	Parasite species	Diagnostic method	Intensity measure
Bakta	1993	Indonesia	2331	hk	KK	EPG
Higgins	1984	Indonesia	227	*asc*, *tri*, hk	MM	EPG
Joe	1959	Indonesia	664	*asc*, *tri*, hk	AUT	Mean worm burden
Margono	1983	Indonesia	276	*asc*, *tri*, hk	KK	EPG
Phongluxa	2013	Lao PDR	574	*asc*, *tri*, hk	KK	EPG (geo mean)
Rahman	1994	Malaysia	204	*asc*, *tri*, hk	KK	EPG
Yogore	1953	Philippines	229	*asc*, *tri*, hk	DM	EPM
Preuksaraj	1983	Thailand	43341	*asc*, *tri*, hk	KK/ST	EPG
Sadun	1953	Thailand	219	hk	DM/ST	EPM
Sadun	1955	Thailand	13469	hk	DM/ST	EPG
Bethony	1998	Thailand	641	hk	NS	EPG
King	2005	Vietnam	201	*asc*	ELISA	EPG
Needham	1998	Vietnam	543	*asc*, *tri*, hk	KK	EPG
Hlaing	1990	Myanmar	2826	*asc*	DM/FEC/KK	EPG
Hlaing	1984	Myanmar	783	*asc*	DM/FEC/KK	EPG
Hpay	1970	Myanmar	571	*asc*, *tri*	DM/FEC/ST	Mean worm burden
Tu	1970	Myanmar	671	*asc*, *tri*	DM/FEC/ST	Mean worm burden

*EPG* eggs per gram, *EPG (geo mean)* geometric mean of eggs per gram, *EPM* eggs per millilitre. *Asc* Ascaris lumbricoides, *tri* Trichuris trichiura, *hk* Hookworm. *KK* Kato-Katz method. *MM* McMaster method. *NS* Not stated. *AUT* Autopsy method. *DM* Direct microscopy method. *ST* Stoll technique. *ELISA* Enzyme-linked immunosorbent assay. *FEC* Formalin-ether concentration method

**Fig. 6:**
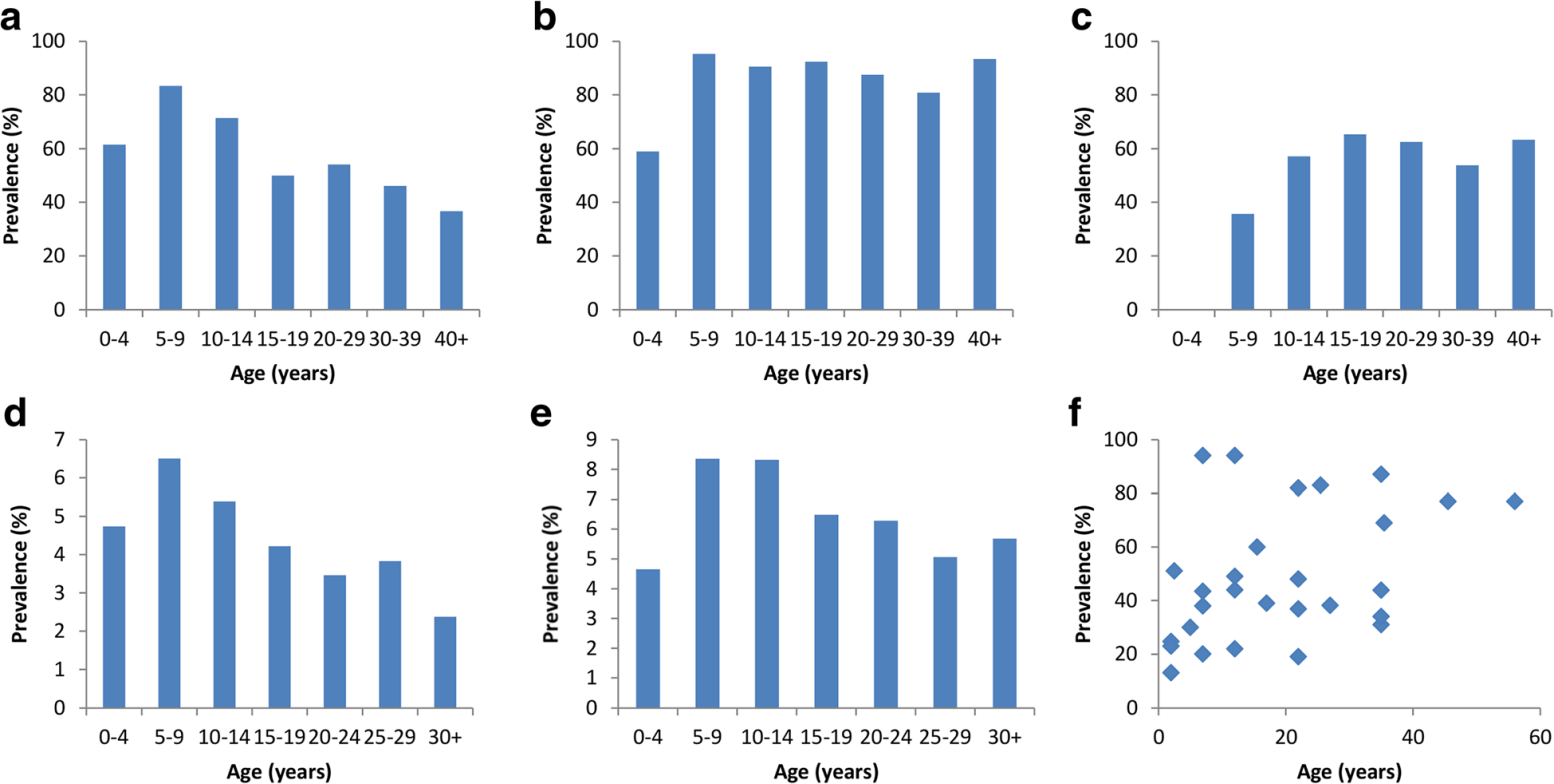
Age distribution of the prevalence of infection for studies in the Philippines (**a**, **b** and **c**) and Thailand (**d**, **e** and **f**) that measured intensity and prevalence across all age groups. Figure (**f**) includes data from more than one study and data points were plotted against the mid-point of the reported age group. A = Philippines, Ascaris lumbricoides. B = Philippines, Trichuris trichiura. C = Philippines, hookworm. D = Thailand, Ascaris lumbricoides. E = Thailand, Trichuris trichiura. F = Thailand, hookworm. Note different scales on figure (**e**) and (**f**)

Figure 6 shows the prevalence age distribution of each STH for two example countries, the Philippines [51] and Thailand [52–55]. Prevalence of *A. lumbricoides* and *T. trichiura* is higher for the included Philippines study (Fig. 6a and b). *A. lumbricoides* prevalence peaks in the SAC groups in both the Philippines and Thailand studies. Hookworm prevalence for both Philippines and Thailand increases to a peak around the 20-29 age groups (Fig. 6c and f), and remains high across the older age groups. However, *T. trichiura* prevalence varies between the two countries; prevalence in the Philippines study is lower for pre-SAC (Fig. 6b) but remains at high level for the other age groups, whereas in the Thailand study (Fig. 6e) prevalence is similarly lower in pre-SAC but then peaks in SAC and decreases over the older age groups. Additional file 1: Figure S1 presents these data for each country included in the review.

**Fig. 7:**
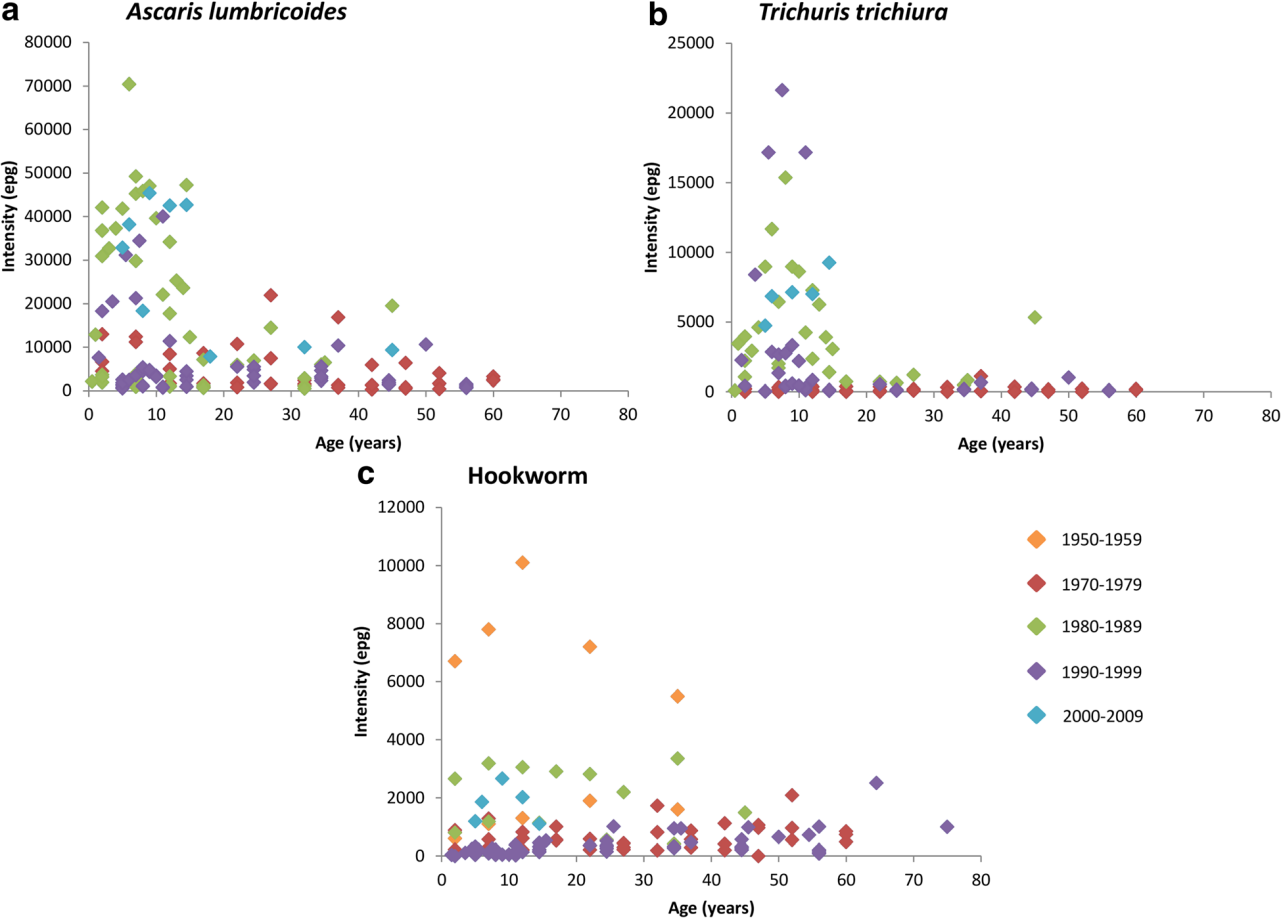
Age distribution of the intensity of infection for studies that measured intensity in eggs per gram (epg), by decade the study was conducted in. **a** = Ascaris lumbricoides, (**b**) = Trichuris trichiura, (**c**) = Hookworm. Orange = 1950–1959, Red = 1970–1979, Green = 1980–1989, Purple = 1990–1999, Blue = 2000–2009. There were no studies from 1960–1969 or 2010-present

Figure 7 records the age distributions of the mean intensity of infection for the studies that recorded faecal EPG, the most commonly used measurement excepting a few studies that employed worm expulsion methods. The plots contain data from studies in all Southeast Asia countries included in the analysis (except for Singapore and Timor-Leste which did not have any studies that included intensity values). *A. lumbricoides* and *T. trichiura* show convex curves with higher mean intensities in the younger age groups (pre-SAC and SAC), which then decreased with increasing age. Hookworm intensities increased with age and then plateaued in older age groups. However, in a few studies there are several data points that denote high intensities in pre-SAC and SAC age groupings. Past comprehensive epidemiological studies have indicated that there is usually a higher intensity of hookworm in adult age groups [21, 50]. A possible reason for this pattern not being seen in this analysis is due to the under-representation of adults in the people sampled in the included studies. For example four studies included hookworm intensity values for SAC [56–59] and none for adults. Also, the age groupings are usually much larger for the adults (e.g. all ages above 40 years or all ages above 50 years) and so may not represent the variation between different adult age groups on a finer age range and the possible increase in hookworm burden in the later years of life. It is relevant to note that very few studies have been conducted post-2000 that record prevalence and intensity of infection across a broad range of age classes – and yet this period to the present is when MDA coverage has been rising. M&E programmes in Southeast Asia, to record the impact of MDA, need to focus on intensity across all age groupings.

**Fig. 8:**
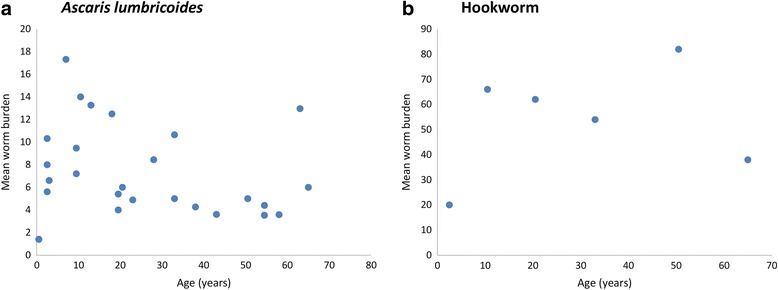
Age distribution of intensity for studies that measured intensity in mean worm burden. **a** = Ascaris lumbricoides, (**b**) = Hookworm

Intensity of infection can also be measured by a more direct method, mean worm burden, based on worm expulsion post-chemotherapy (Fig. 8). As this method directly counts the number of worms harboured by a participant, there is perhaps little uncertainty of the person’s intensity level, assuming that stool collection is complete and lasts for many days post-treatment. However, there are far fewer studies that use mean worm burden as the intensity measure as well as reporting these intensities by age and including a representation of the entire community [60–63]. All but one of these studies are in Myanmar. The remaining study [60] was carried out in Indonesia and was the only case in this review to collect data via autopsy. The data for *T. trichiura* was not included in Fig. 8 because, in two studies, the adult age groups were grouped in a manner that does not adequately reflect the variation across adult groups (all ages above 14 years). For *A. lumbricoides* mean worm burden has a small peak around SAC, decreases around 30–50 years old and then increases again at the oldest ages (Fig. 8a). Therefore, in terms of worm intensity, the older age groups also suffer from a high worm burden, although the morbidity in adults may be less damaging than in SAC. However for hookworm, for which there is only one study [60], mean worm burden peaks in the 30–40 years individuals (Fig. 8b).

### Limitations

The first limitation of our review, which has been highlighted earlier in this paper, is our inability to directly compare studies due to the differences in diagnostic techniques and units of measurement. However, despite this lack of standardisation, when the studies were pooled and analysed, trends in prevalence and intensity across the age classes could be determined. The age distributions of *A. lumbricoides*, *T. trichiura* and hookworm prevalence (Fig. 6) displayed a similar pattern to those recorded in past studies and reviews [46, 50, 61, 64]. However, in future research and in M&E appraisals it is obviously highly desirable to place greater emphasis on the standardisation of the methods of measurement.

**Fig. 9:**
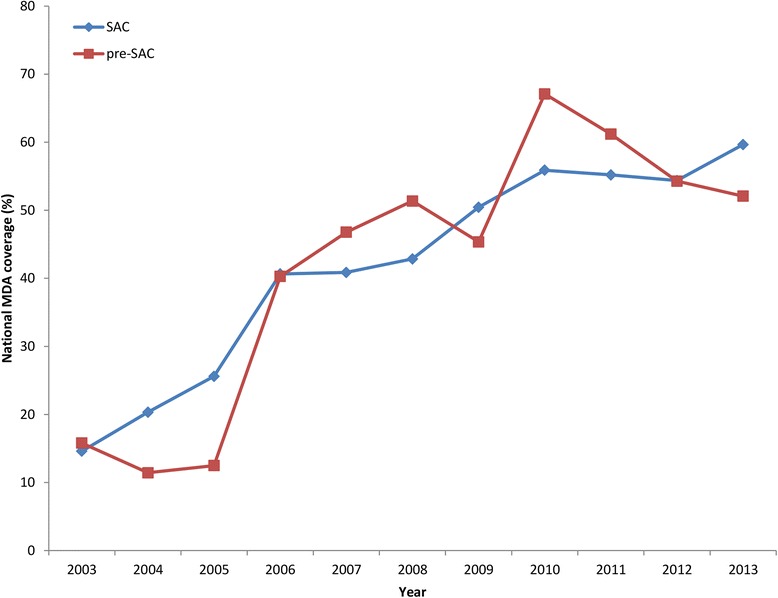
Mean mass drug administration (MDA) coverage for pre-school aged children (pre-SAC) and school-aged children (SAC) in the Southeast Asian countries. Red = pre-SAC, Blue = SAC. Data collated from the World Health Organization preventive chemotherapy databank [20]. Country-specific data is included in Additional file 3

Secondly, the significance of changes in the prevalence and intensity of STH infection over time can best be interpreted when examined in conjunction with knowledge of the history of control efforts, specifically MDA, in the area under consideration. It is possible to determine this history at country-level via the WHO PCT databank [20], but the data only extends as far back as 2003 and is incomplete for a number of countries. Regional-level MDA data and information on other interventions, such as water and sanitation hygiene (WASH) and behaviour change, is required before the cause of any changes in prevalence and/or intensity can be established. Figure 9 plots the average (over the reviewed Southeast Asian countries) change in MDA coverage as recorded in the WHO PCT database in pre-SAC and SAC. The raw data, as extracted from the WHO PCT databank interactive report, is presented in Additional file 3. Note that average coverage increases from 2003 to 2013 in both age groupings as a trend in the region. However, considerable differences exist between countries as recorded in the raw data. It seems likely that this overall rise in coverage is the main driver behind the trend for a decrease in prevalence and intensity of STH infection in most countries in the region.

Finally, whilst every effort has been made to make this review comprehensive and inclusive of all the studies published on STH in Southeast Asia, it is inevitable that some studies will have been missed due to them not being published in English language journals. In the future, it would be useful to have these papers translated to add further information to the review. It is likely however, that the inclusion of these papers would have supported the conclusions drawn in this paper. In general, STH studies are difficult to compare and contrast due to a lack of standardisation in diagnostic methods.

## Conclusions

Since 1947 there have been a large number of epidemiological studies on STH in Southeast Asia, increasing in numbers over the years to the present day. However, only a small proportion of these studies can be truly deemed comprehensive in the sense of good coverage of prevalence and intensity data over a broad range of age classes in the studied communities or areas. Only 17 studies (6 %) measured both STH prevalence and intensity across all age groups. It is imperative for the future of STH control in Southeast Asia that more studies are completed with much better, standardised M&E design. Ideal M&E design should include a consistent diagnostic method. Currently Kato-Katz is recommended by the WHO due to its ease of use in the field and low cost [19]. However, methods with higher sensitivity and specificity are desirable as the impact of expanded MDA programmes drive infection to low levels. M&E should use STH intensity as the primary measure to determine the effectiveness of MDA programmes, including those integrated with WASH, education and nutrition components. Large changes in intensity, reflecting effective treatment, are not well measured by changes in prevalence. M&E should also cover the different ecological areas of a country and should be repeated at set intervals. They should be continued for a specified time after MDA, and any other control activities, have been stopped. The data is of obvious importance in evaluating the progress and effectiveness of MDA programmes, plus the impact of treating pre-SAC and SAC on transmission within the total community. Finally, this data can also be of great value in mathematical modelling studies to determine the most effective targeting by age group and frequency of MDA to interrupt transmission of STH in different populations and settings.

Perhaps the most important message arising from this review is that public health workers must place greater emphasis on the standardisation of diagnostic methods for determining STH prevalence and intensity. The achievement of some degree of uniformity in epidemiological methods would mean that studies can be directly compared both within and between countries to effectively evaluate and compare the progress of control efforts. To achieve this, guidance and support from organisations such as WHO to endemic countries would be useful for emphasising the importance of comprehensive and continuous M&E. Indeed it would be desirable if WHO played the lead role in setting standardisation guidelines. Training in best practice and uniform methods could help lower income countries optimise M&E. It has proven difficult in the past for researchers to agree on one method of STH diagnosis. There have been numerous recent papers evaluating the sensitivity and specificity of the various methods [30, 65, 66]. However, the new technologies of quantitative PCR, multiplex PCR and ELISA offer great potential especially in low prevalence areas, as MDA impact increases, to identify the remaining pockets of transmission.

## Additional files

Additional file 1:
**PRISMA checklist, full list of search terms and Supporting Figure 1.** (DOCX 1462 kb) Additional file 2:
**Full list of the included studies.** (XLSX 56 kb) Additional file 3:
**Preventive chemotherapy data for the Southeast Asian countries.** (XLSX 13 kb)

## Abbreviations

Asc: *Ascaris lumbricoides*
ASEAN: Association of Southeast Asian Nations
AUT: autopsy method
CDSR: Cochrane Database of Systematic Reviews
DM: Direct Microscopy method
ELISA: enzyme-linked immunosorbent assay
EPG: eggs per gram of faeces
EPG (geo mean): eggs per gram of faeces (geometric mean)
EPM: eggs per millilitre of faeces
FEC: formalin-ether concentration
GAHI: Global Atlas of Helminth Infections
Hk: hookworm
KK: Kato-Katz method
Lao PDR: Lao People’s Democratic Republic
M&E: monitoring and evaluation
MDA: mass drug administration
MM: McMaster method
NS: not stated
NTD: neglected tropical disease
PCR: Polymerase Chain Reaction
PCT: preventive chemotherapy
Pre-SAC: Preschool-aged children
PRISMA: Preferred Reporting Items for Systematic Reviews and Meta-analyses
SAC: school-aged children
SE Asia: Southeast Asia
ST: stoll technique
STH: soil-transmitted helminth
Tri: *Trichuris trichiura*
WASH: water and sanitation hygiene
WHO: World Health Organization
