# Supplementary material for: Epidemiological surveys of, and research on, soil-transmitted helminths in Southeast Asia: a systematic review
Source: Parasit Vectors. 2016 Jan 27;9:31. doi: 10.1186/s13071-016-1310-2 (PMC4728827; doi:10.1186/s13071-016-1310-2)
Supplement: Additional file 1: — PRISMA checklist, full list of search terms and Supporting Figure 1. (DOCX 1462 kb) [file 13071_2016_1310_MOESM1_ESM.docx]

Epidemiological surveys of, and research on, the soil-transmitted helminths in Southeast Asia: a systematic review

**Additional File 1**

**PRISMA Checklist**

**
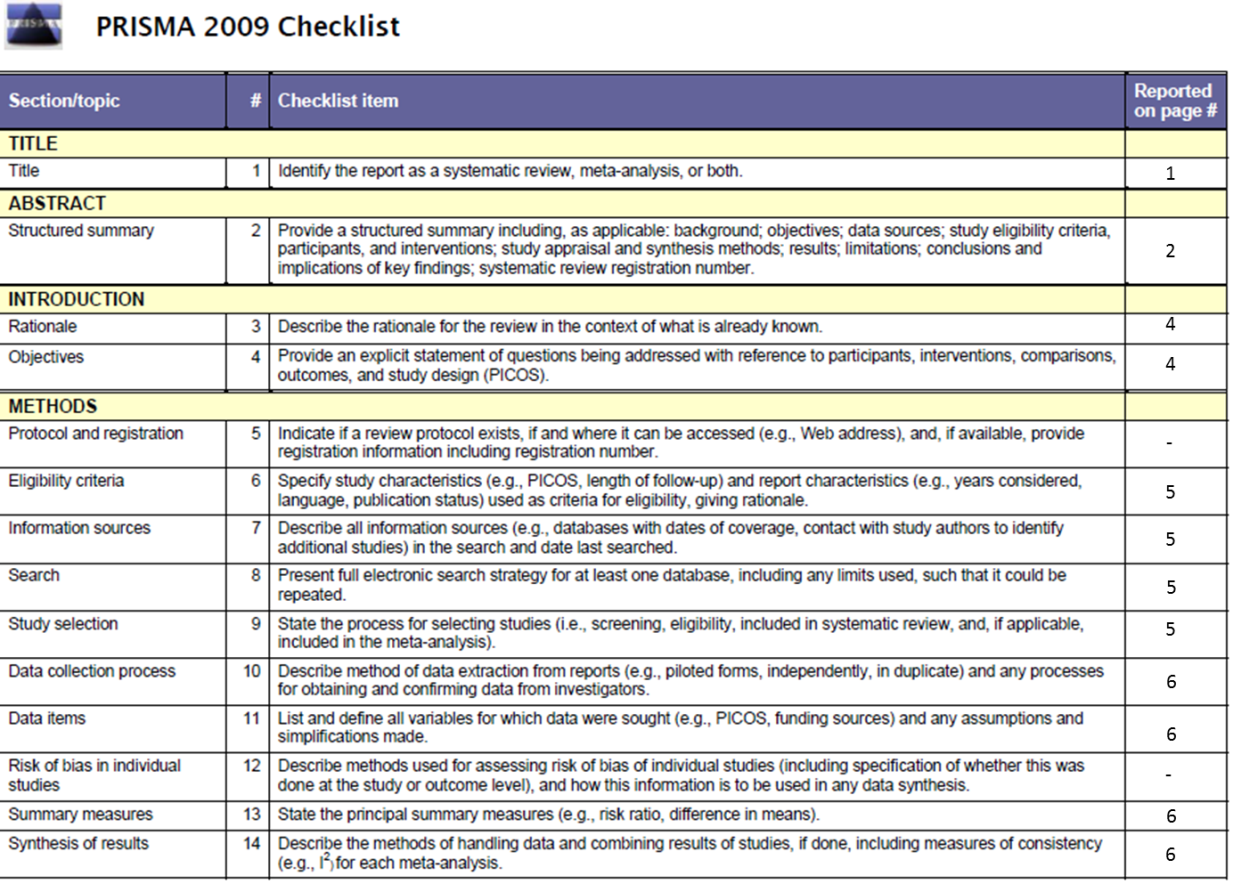
**

**
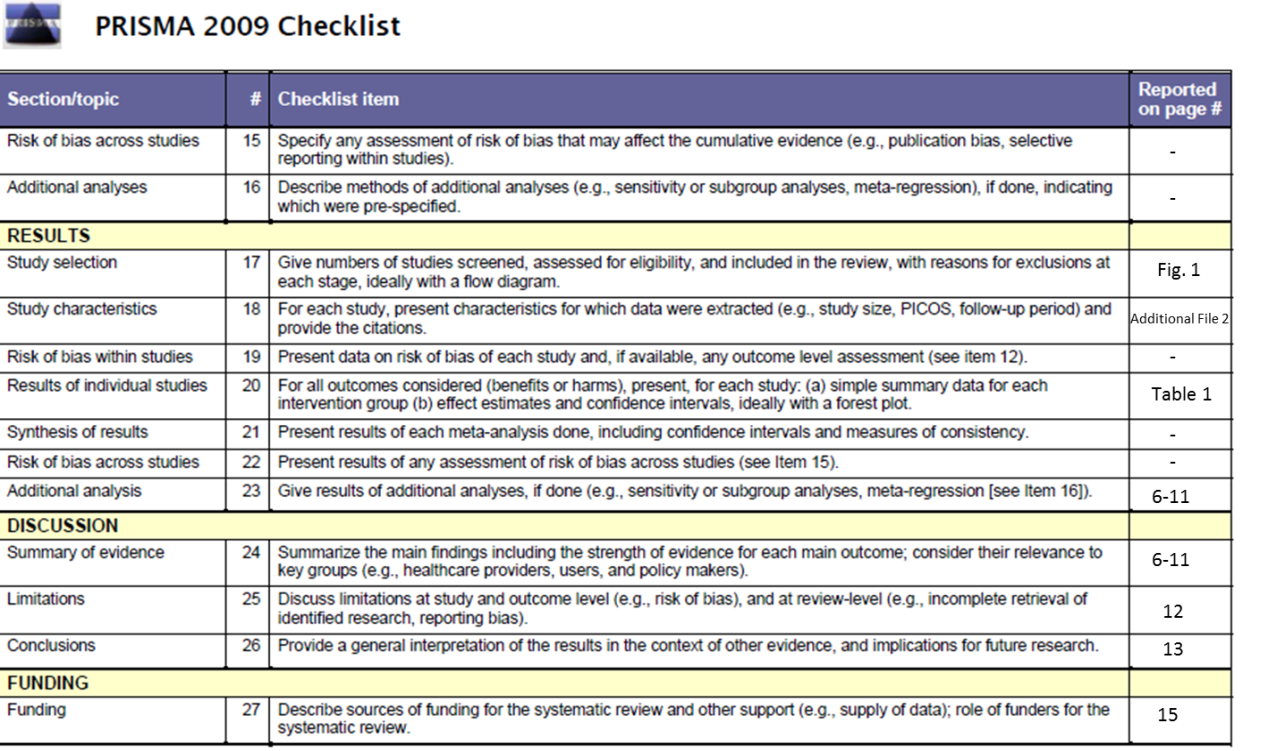
**

**Full list of search terms:**

*sth*

*soil-transmitted helminth*

*ascaris*

*trichuris*

*hookworm*

*helmint**

*parasit**

*necator*

*ancylostoma*

*deworm**

*(country name)*

NOT *plasmodium*

**
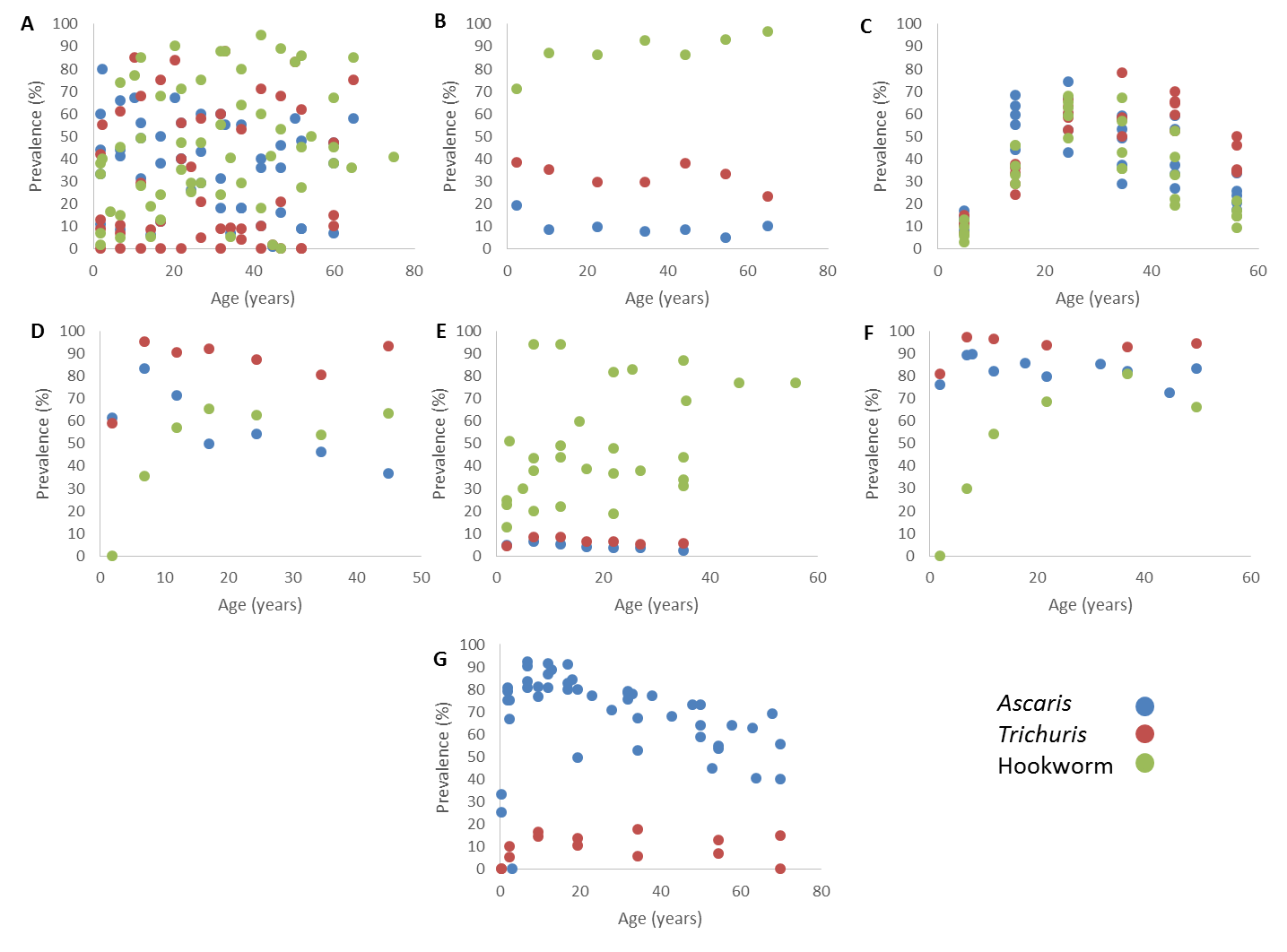
**

**Supporting Figure 1: Age distribution of prevalence for studies that measured intensity and prevalence across all age groups.** *Data points were plotted against the mid-point of the reported age group. A=Indonesia, B=Lao PDR, C=Malaysia, D=Philippines, E=Thailand, F=Vietnam, G=Myanmar. Blue=Ascaris lumbricoides, Red=Trichuris trichiura, Green=Hookworm.*
